# Numerical study on effect of constant and variable axial loads on RC column subjected to blast load

**DOI:** 10.1016/j.heliyon.2022.e11681

**Published:** 2022-11-19

**Authors:** Solomon Abebe, Solomon Getachew

**Affiliations:** Department of Civil Engineering, Debre Markos University, Ethiopia

**Keywords:** Blast load, Constant axial load, RC Column, Variable axial load

## Abstract

Explosion is an instant release of potential energy, heat, temperature and sound producing a pressure wave that travels away from the source radially on which the resulting force the blast wave is a blast load. Besides this, column especially external column as a main load bearing compressive structural component in buildings is the most critical structural element vulnerable to explosion and require an in-depth investigation on its performance against blast loads. Despite this, currently there is perceived gap and meagre of researches regarding numerical investigation on effect of presence of constant and variable axial loads on reinforced concrete column when subjected to blast load. In this study, the responses of reinforced concrete column under different scaled distance blast load scenarios accompanied by constant and axial loads were investigated. The finite element method of structural analysis was employed accompanied by nonlinear explicit time integration software LS-DYNA. The numerical analysis result revealed that RC columns subjected to large axial loads accompanied by small scaled distance blast scenario made the reinforced concrete column to suffer severe damages including crushing of concrete especially direct shear failures and breaking of reinforcing steel bars. In addition to this, comparing the nature and behaviour of variable axial loads with constant axial loads, the former loading case revealed larger nodal displacement values along height of column, and a higher displacement-time history curve was traced. On the other hand, the damage propagation nature of RC columns loaded with variable axial load was slow and progressive and different with RC columns with constant axial loads accompanied by small scaled blast scenario which was rendered to have a severely crushed concrete element without bending actions leaving the entire column inadequate for service.

## 1 Introduction

Explosion is an instant release of potential energy, heat, temperature and sound producing a pressure wave that travels away from the source radially. The energy sources accountable for the detonation can be chemical, mechanical, nuclear, or electrical. The principal effects of the explosive output are blast induced overpressures, fragments generated by the explosion and the shock induced loads produced by the shock wave transmitted to a medium such as air and/or ground. Of these three parameters, the blast pressures (incident overpressure and/or reflected) overpressures are usually the governing factor in determination of structure responses [1, 2].

Blast by definition, it is a transient change in the gas density, pressure, and velocity of the air surrounding an explosion. Whereas blast load is the load resulting from the acting forces (overpressure and drag forces) from the blast wave. At a given detonation start point, the detonation wave rapidly converts the solid or liquid explosives into a very hot, dense, high-pressure gas, and the volume of this hot expanded gas which was an explosive material before the detonation is then the source of strong blast waves in air which are then composed of a high intensity shock front which expands outward from the surface of the explosive into the surrounding air in radial wave form. As the wave expands, it exponentially decays in strength, lengthen in duration, and decrease in velocity DOD [1].

On explosion related incidents, the most significant feature is the sudden release of energy to the atmosphere which results a blast wave. Depending upon magnitude of rise of pressures blast wave can be classified into two namely shock and pressure waves. Shock waves are characterized by a sudden rise in pressure above the atmospheric ambient pressure to a peak incident pressure. Since the sudden rise of pressure above ambient pressure creates a push over action on existing air field thus creates a partial vacuum of air space behind the shock front faces which then creates a suction action creating a negative phase of a pressure wave, after all this pressure disturbance gradually returns to nature's state of equilibrium. On contrary, if the pressure has a gradual rise to the peak incident overpressure followed by a gradual pressure decay and a negative phase of pressure loading similar to that of the shock wave it is termed as pressure wave ASCE [2].

For a blast resistant structures, the principal parameters of the blast wave required to define the blast loading of a building's components are (a) Peak side-on positive overpressure, P_so+_, positive phase duration, t_d+_, and the corresponding positive impulse, I_o+_. (b) Peak side-on negative overpressure, P_so−_, negative phase duration, t_d−_, and the associated negative impulse, I_o−_ ASCE [2].

**Figure 1:**
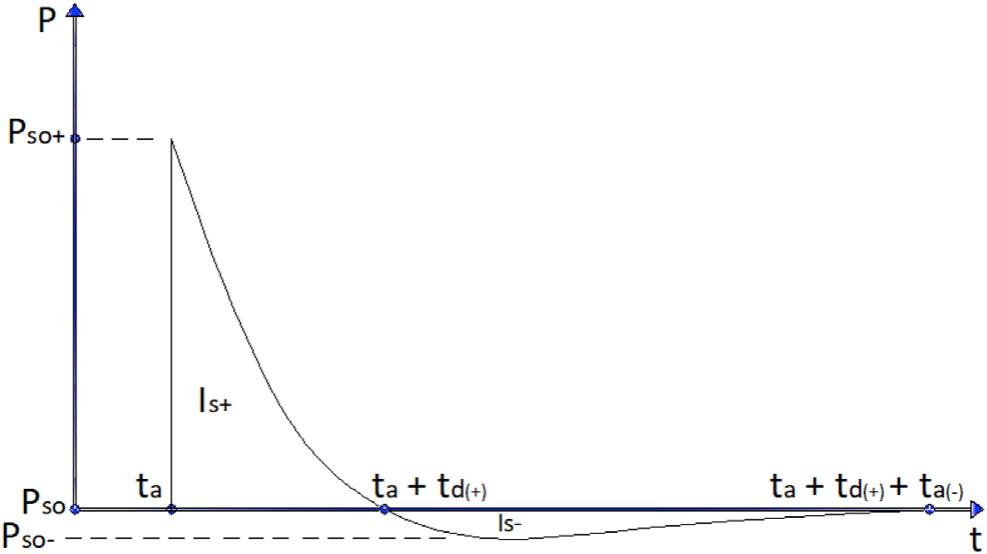
Characteristics shape of blast induced shock wave.

The ideal characteristic shape of shock wave is shown in Figure 1. Peak side-on positive pressure (P_so+_) is the incident pressure on which it is a state of pressure in undisturbed flow field. Peak side-on negative pressure (P_so−_) is the maximum value of the overpressure on particular negative phase of a pressure. The time required to reach a blast induced shock wave to a structure is time of arrival (t_a_). The duration of a blast wave profile where pressure is above and below ambient pressure is termed as positive and negative phases respectively. Positive and negative specific impulse (I^+^ and I^−^) is the integral of the positive and negative pressure-time curve generated by an explosion source respectively.

Blast pressure, load duration, impulse shock wave velocity, arrival times, and other blast parameters are usually presented in scaled form. The most common form of scaling is called “cube root scaling (Z)” owing to the fact that blast parameters are scaled by the cube root of the explosion energy (see Eq. (1)).

(1) $$Z=\frac{R}{W^{\frac{1}{3}}}$$

where, *R* is the distance from the center of explosion and *W* is the charge mass.

Likewise, the use of TNT as the ‘reference’ explosive is the other way of scaling a blast induced shock wave parameters. The simplest way of evaluating the TNT equivalence of a specific explosive is to multiply the mass of explosive by a conversion factor based on its specific energy and that of TNT Smith [3].

Studies on dynamic behaviour, nature of propagation, and damage mitigate techniques have been conducted on columns especially external columns which are more vulnerable to explosion. Siba [4] experimentally investigate response of RC columns under near-field blast loading events. The experimental program was carried out in open field arena at the Canadian forces base (CFB) Petawawa, Ontario. On this study the effects of near-field explosions on reinforced concrete columns with different transverse reinforcement detailing and at different scaled distances (z = 0.22 m/kg^1^/3, z = 0.54 m/kg^1^/3, and z = 0.86 m/kg^1^/3), different charge mass (100 kg and 150 kg) using ANFO explosives, Axial force ratio (0 and 0.3) were also experimentally investigated. From the experimental study results (Siba) claimed that scaled distance and axial load ratio had a significant effect on the response of RC column against blast load.

[5, 6, 7, 8] have performed analytical studies by using a SDOF and MDOF structural dynamic analysis to study the response of reinforced concrete columns under blast loads. In addition to this a numerical assessment on RC columns prone to blast loads are also evaluated by [9, 10, 11, 12, 13, 14, 15, 16, 17]. [9, 10] evaluated the damage propagation of RC column strengthened by steel jacketing and also predicted the residual strength of a FRP retrofitted RC column subjected to blast loading. Thai et al. [9] investigated the effect of different loading levels on the residual capacity of retrofitted RC column. From the study results, it was revealed that loading level had significant effect on the dynamic response of a column. Likewise Thai et al. [10] also considered different compressive axial load ratios and investigated the damage modes of RC column. A compressive axial load ratio of 0.0–0.8 f_c_′A_g_ were considered and the results showed that as the scaled distance drops, the effect of increasing an axial load made the column in severe damage.

Gholipour et al. [11] conducted a study on axially loaded RC columns subjected to the combination of impact and blast loads. Explicit time integration algorithm-based software LS-DYNA was deployed. In this study, a specific assessment on vulnerability of the RC column to sever loading-related parameters including the loading sequence, time lag, axial load ratio, loading location, and impact velocity were discussed. The study result from the FE simulation showed that the combination of a middle-rate impact loading and a close-in explosion provided more intensive loading conditions when they apply at the same elevation on the column. Consequently, *Zadeh* [12] conducted finite element analysis to study the pressure-impulse diagrams for reinforced concrete columns subjected to blast loading. The nonlinear response of reinforced concrete structures when subjected to impact and blast loading was conducted in accordance with two levels: material level and structural level. At the material level, the strength enhancement of three material models of LS-DYNA subjected to high strain rates was evaluated. At the structural level, Pressure-Impulse diagrams for reinforced concrete columns that have four configurations of transverse reinforcement were developed. Finally, the key findings of this study forwarded a remarkable output on the effect of aspect ratio, number and area of longitudinal reinforcement, compressive strength of concrete, and axial service loads.

Rajkumar et al. [13] reported a numerical study result for reinforced concrete column under blast loading with sole objective of quantitatively investigating the effect of geometry on RC columns under a variability of blast loading. Explicit time integration FE code software LS-DYNA and a parametric analysis on different shapes of columns was employed. Finally, a result from FEA showed that the reinforcement ratio, seismic detailing, and scaled distance had great influence on the blast performance of RC columns.

Abladey [14] performed a numerical assessment on reinforced concrete column under close-in blast load. The researcher deployed a nonlinear explicit time integration based software (AUTODYN) and a result from the numerical analysis revealed that the natural period of vibration of reinforced concrete columns surged with a decrease in scaled distance. Furthermore, the effect of tie spacing played a significant role in mitigating damage. Besides this, the researcher forwarded a remarkable point to investigate the effects of axial load ratios on the response of reinforced concrete column prone to blast induced shock wave. Likewise, the effects of different reinforcement scheme and column shape on the response of RC columns subjected to blast loading was also conducted by Patil [15]. RC columns were detailed according to IS 456:2000 and IS 13920:2016 Indian Standard Codes. Using a high-fidelity physics-based FE code, LS-DYNA V971. The damage mitigation capability of different reinforcement schemes and column shape on blast resistance of RC column was evaluated. The numerical analysis result revealed that the transverse reinforcement spacing significantly affects the behaviour of RC column when subjected to blast loading. The numerical analysis result also revealed that circular shaped columns displayed prominently higher blast resistance when compared to square shaped columns.

Further study on the vulnerability and damage analysis of reinforced concrete frame buildings subjected to near field blast events was performed by Jayasooriya [16] and an investigation on the response of reinforced concrete (RC) framed buildings together with their load bearing key structural components (columns) to a near field blast event was presented. Jayasooriya [16] employed a Finite element method (FEM) based analysis and examined the structural framing system and components for global stability, followed by a rigorous analysis of key structural components (columns) for damage evaluation using the codes SAP2000 and LS-DYNA V971 respectively. As a remark, Jayasooriya [16] presented two types of design methods, i.e. RC columns detailing with multi-layer steel reinforcement cages and a composite column including a central structural steel core to avoid complete collapse of framing system.

Rajagopal et al. [17] studied the plasticity-based material model for concrete subjected to dynamic loadings. This study proposed a new plasticity-based material model named UMAT, which had three parts with discussion on equation of states, damage definition, and a definition on the modified strength surface. The development and implementation of a user defined material model was executed by using LS-DYNA software. In addition, a parametric study was performed with the proposed by varying the thickness of an element reinforcement ratio, scaled distance, and concrete strength. It was found that scaled distance played a significant role in influencing the blast analysis results.

Axial loads which are known to be induced into a column can be classified into two clusters namely constant and variable axial loads. The constant axial loads are known to be induced by the weight of the supported mass over the period of time. On the other hand, variable axial loads are assumed to be induced from the dynamic reactions of framed structural member caused by the effect of the blast load on the supported mass. The aforementioned current study trends and efforts focused on a lot of research works on the response of reinforced concrete column subjected to blast induced shock waves. As mentioned in the previous section, even though some researchers employed different analysis techniques, the analysis was limited to induce axial loads by a constant axial load time steps which showed that, the variable axial loads were omitted. Thus, in this study, since the FE simulation and analysis considering the effect of different type of loadings including constant and variable axial loads under blast induced shock waves are not yet available on current knowledge, this study investigated a numerical study on RC columns subjected to different level of axial load in constant and variable time step, extracted the post-processed data in terms of displacement-time history, displacement along the height of column, and effective plastic strain damage profiles, this study bridges the knowledge gap on the study of RC column subjected to different level of axial loads with distinct loading time step.

## 2 Finite element analysis modelling of RC column

### 2.1 Modeling of concrete

**Figure 2:**
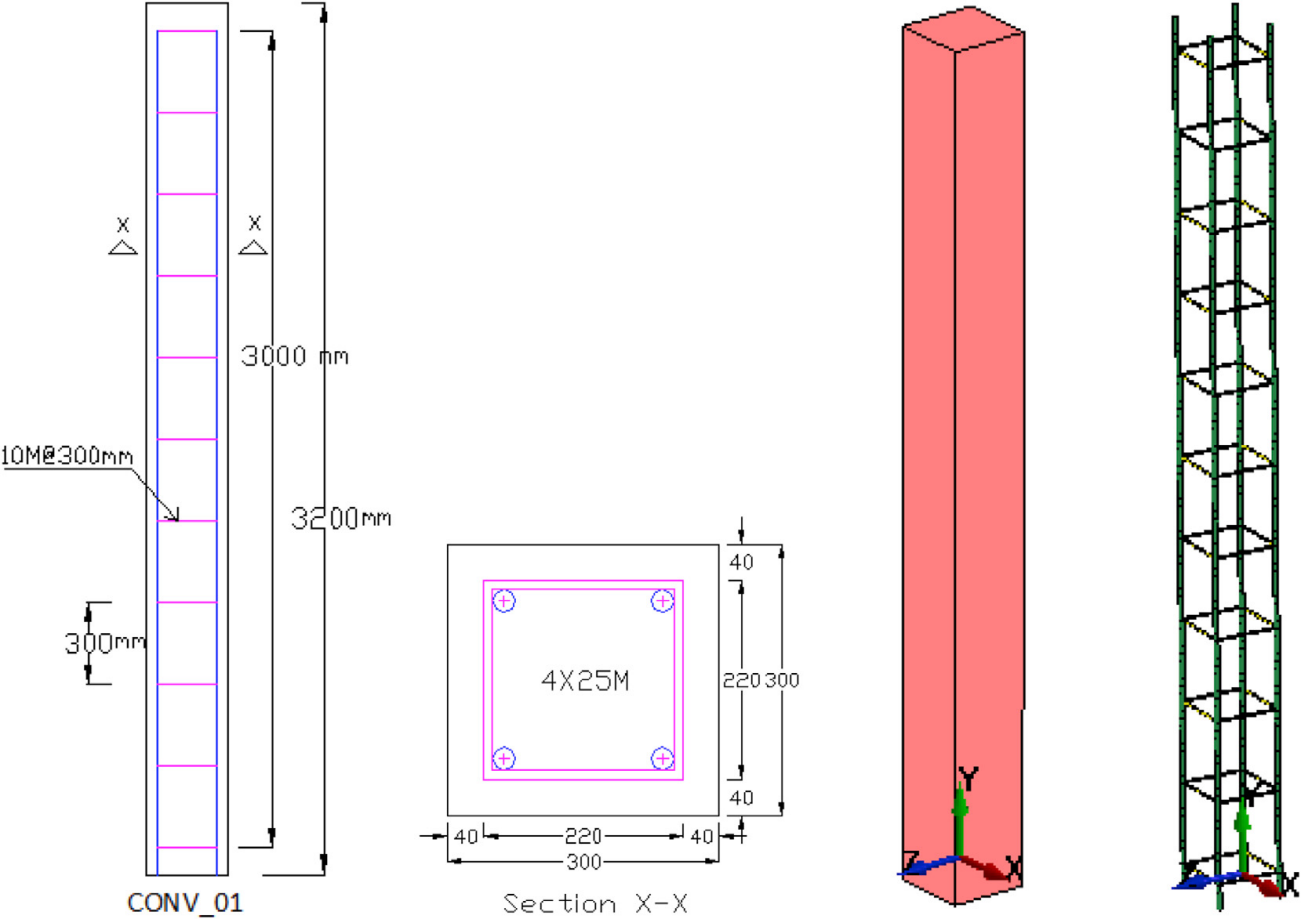
RC column solid and reinforcing steel bar elements.

The concrete part of the column modeled as solid element comprising a 300 mm × 300 mm transverse cross section dimension and 3200 mm height (see Figure 2). A constant stress solid section element and a truss element was defined for solid and beam element respectively.

∗Mat_159_Continous_Surface_Cap_Model material model is an enhanced concrete model designed for blast and impact loading systems and was used for solid element assignment. The behaviour of this material can be controlled by the following user specified variables: unconfined compressive strength (FPC = 40.00 MPa), concrete mass density (RO = 2.70e−09 tonne/mm3), maximum aggregate size (DAGG = 19.00 mm), strain rate (SRATE = ON), damage functions in terms of (ERODE = 1.05) option, and UNITS = 7 on which the keyword card uses EQ.7: metric ton, millimeter, second, MPa unit conversion flag were fairly enough to present an outline of a material model that should yield reasonable results.

### 2.2 Modelling of reinforcing steel

Reinforcing steel carries either tensile or compressive stresses. Thus, both longitudinal and transverse steel reinforcing steel bars were modeled by truss element formulation. The obligatory input variables for the ∗SECTION_BEAM for both longitudinal and transverse rebars includes section id. (SECID), element formulation key (ELFORM) and cross-sectional area (A).

∗MAT_003R3_ELASTIC_PLASTIC_WITH_KINEMATIC_ISOTROPIC material model was used for both longitudinal and transverse rebar element assignment. By varying the value of β between 0 and 1, ∗MAT_003 material model generation module has the ability to create kinematic hardening effects. In addition to this, the behaviour of this material model is controlled by user defined yield tensile stress (SIGY = 400.00 MPa), steel mass density (RO = 7.80e-09 tonne/mm3), steel elastic modulus (E = 2.1e+5 MPa) and poisons ratio (PR = 0.3) were enough to present a reasonable results.

### 2.3 Boundary and loading conditions

The boundary condition of the column was defined by using a Single Point Constraint (∗SPC) system. Besides this, three loading conditions namely self-body-weight, axial and blast loads were imposed into the system (RC column) by using ∗LOAD_BODY, ∗LOAD_BEAM and ∗LOAD_BLAST_ENHANCED respectively.

**Figure 3:**
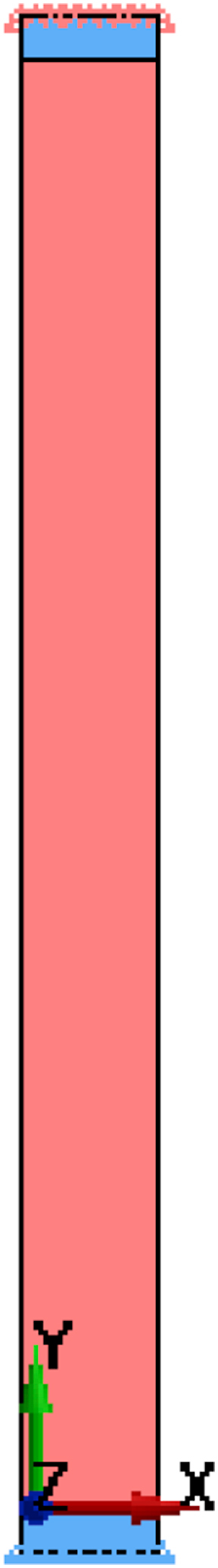
Sample ∗SPC fixed-fixed support system.

A Single Point Constraint (∗SPC) system that constraints nodal displacements of a system was deployed and for the case where axial loads were supposed to be induced into the system, keeping the bottom support conditions fixed, the top nodes of the system were allowed to release the vertical translational restraint system so that an axial strain due to vertical axial loads can easily induced into the system. This all support condition variation was implemented by using a 1 and 0 ∗SPC_SET keyword command on which input value of 0 release and 1 restraint nodal displacement (translation and rotation). Figure 3 illustrates sample single point constraint system for fixed-fixed support system.

**Figure 4:**
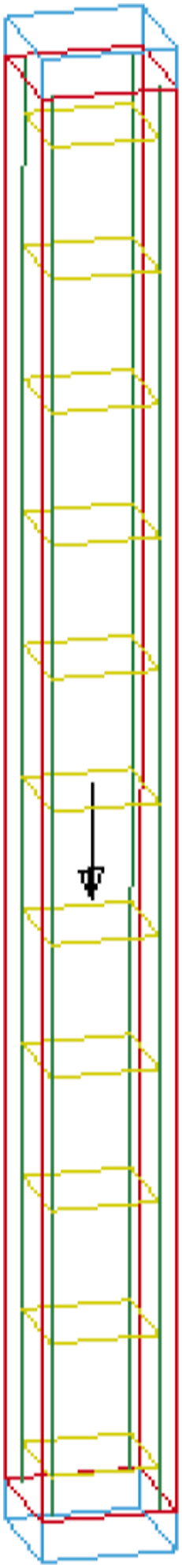
LS-DYNA ∗LOAD_BODY keyword card output.

The body weight (Figure 4) of the column was taken into account by using ∗LOAD_BODY command and gravity scale factor of (SF = - 0.00981), which is usually applicable for determining the load curve was deployed.

**Figure 5:**
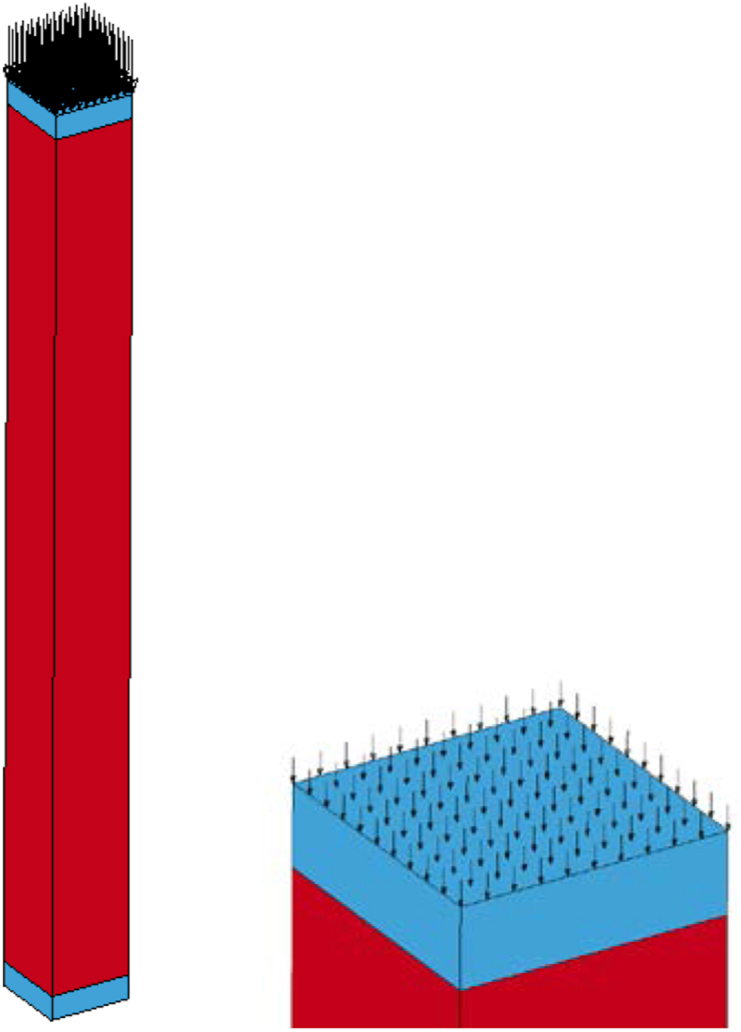
LS-DYNA ∗LOAD_BEAM keyword card output.

To avoid local stress concentration on the column, the axial load which was supposed to be imposed into the system was allowed to be uniformly distributed into the top nodes of the cover plate (Figure 5). After employing a load per node system, the ∗LOAD_BEAM command was activated by using necessary inputs including direction of applied load (DOI), specified load curve id (LCID) and scale factor (SF = −1). While doing so, the negative sign represented the downward direction of the applied load.

**Figure 6:**
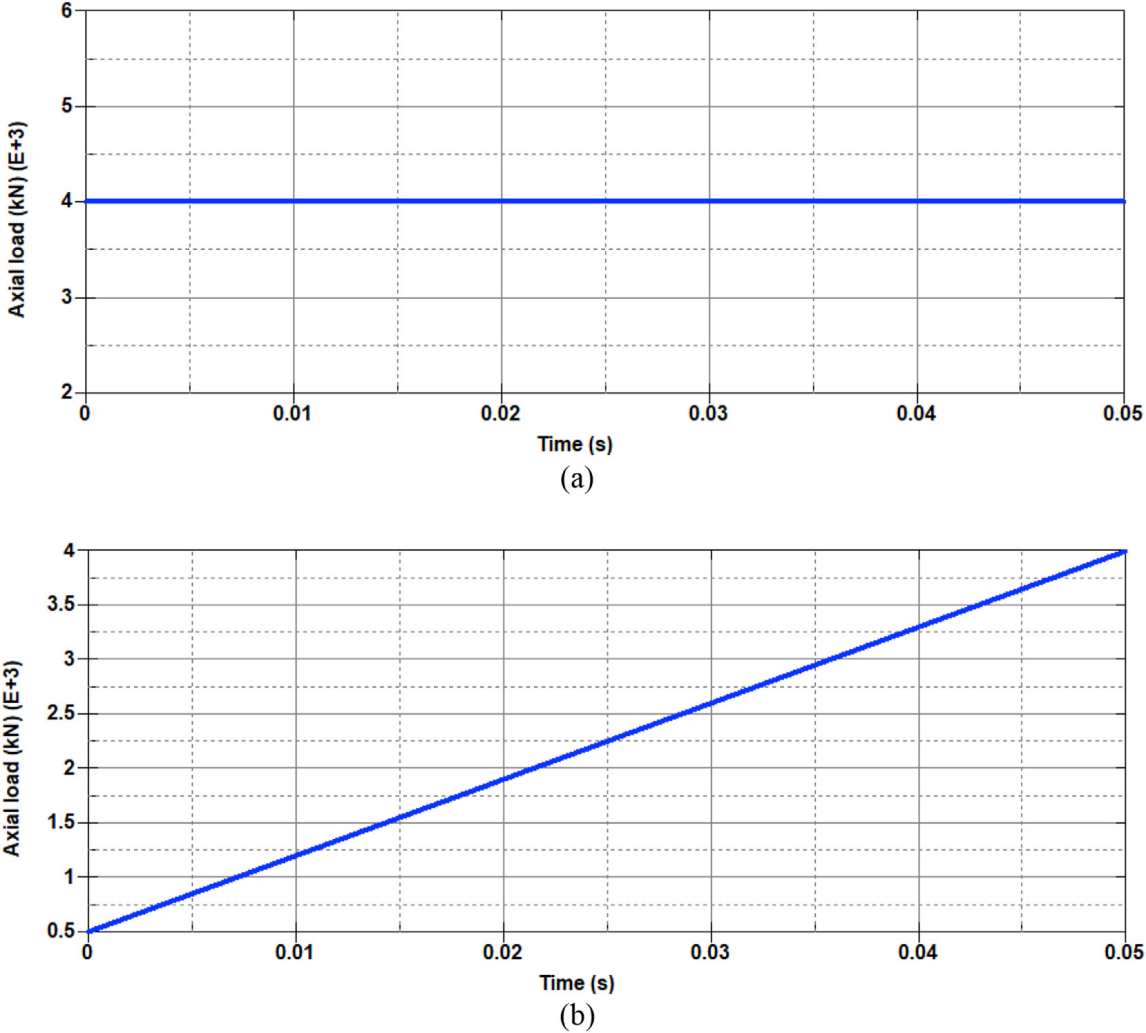
Sample factored axial load curve for 0.30 ALR: (a) constant and (b) variable.

Consequently, the effect of variation in constant and variable axial loads was imposed into the system by using a DEFINE_CURVE key word. As depicted in Figure 6a and b, RC columns with constant and variable axial loads, load curves with static and linearly varying magnitude of axial loads were defined. Figure 6a depicts the way how a 4000 kN constant axial load was imposed into the RC column for a 50 ms analysis time. Likewise, to avoid impulsive application of a compressive strain, a linearly variable axial load was imposed into the system by incorporating a gradually increasing load curve (see also Figure 6b).

On the other hand, the blast load was defined by using a ∗LBE keyword command which uses empirical data from CONWEP to explosives in conventional weapons. By defining explosive's charge center (XBO, YBO and ZBO), unique blast id (BID), type of blast source (BLAST) and unique segment set ∗LBE keyword file module applies a blast function for the application of pressures.

### 2.4 Contact algorithm

**Figure 7:**
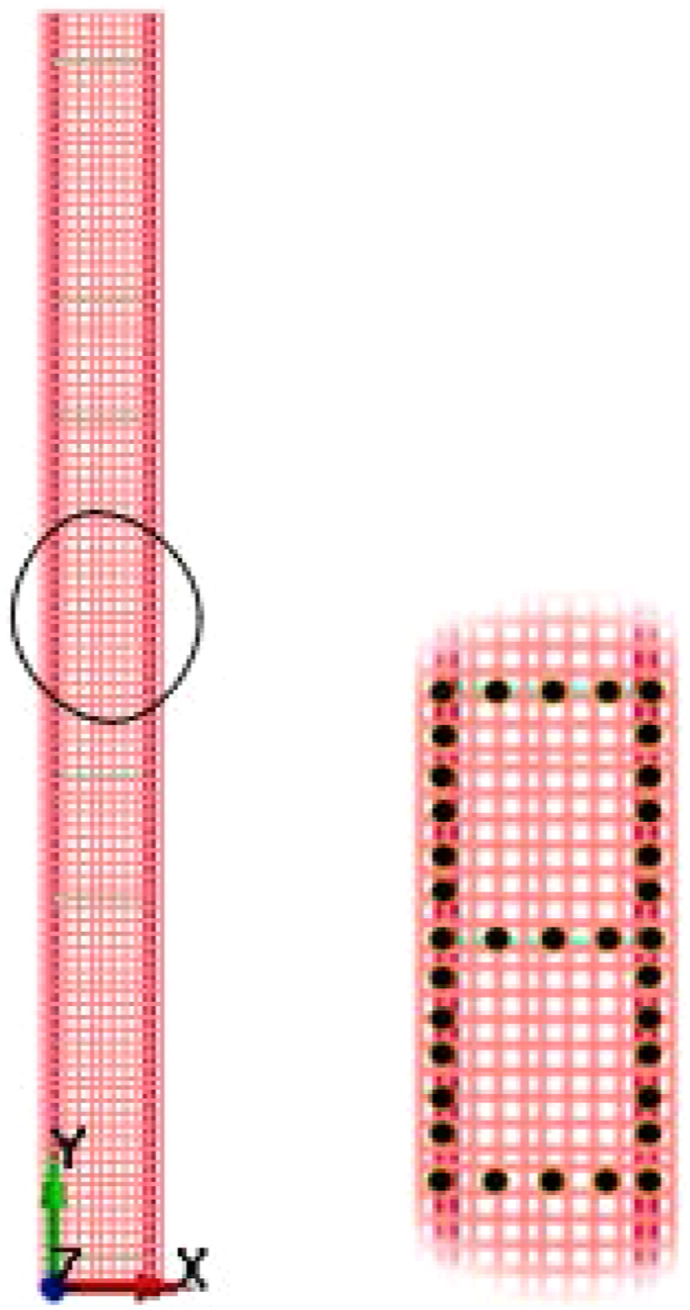
Concrete steel reinforcement ∗ALE surface contact.

To create a bond between concrete and steel reinforcement, an AUTOMATIC GENERAL, ALE and GENERAL TRANSDUCER PENALTY algorithm-based interaction which further required determination of a slave and master parts were used. While doing so, a slave and master definition for steel reinforcement and concrete elements were assigned respectively. By doing so, the steel reinforcement and concrete elements were allowed to share nodes to achieve a fully coupled interaction (perfect bond) with no slip. Figure 7 shows a typical surface contact plot for study RC column.

### 2.5 Solution control

To create a stable solution on energy control, the solution controls including energy, hourglass, termination time and time step keywords were defined. For hourglass viscosity type a typical standard type, specifically a standard LS-DYNA with 0.1 hourglass coefficient which was sufficient for stability checkup was used. On the other hand, t run time limit of 50 ms was used in the analysis termination time keyword catalogue. The initial time step size was permitted to be determined by LS-DYNA analysis module. A 0.9 scale factor for computed time step with a characteristic length = area/longest size basic time step size computation algorithm was used.

### 2.6 Displacement monitoring gauges

**Figure 8:**
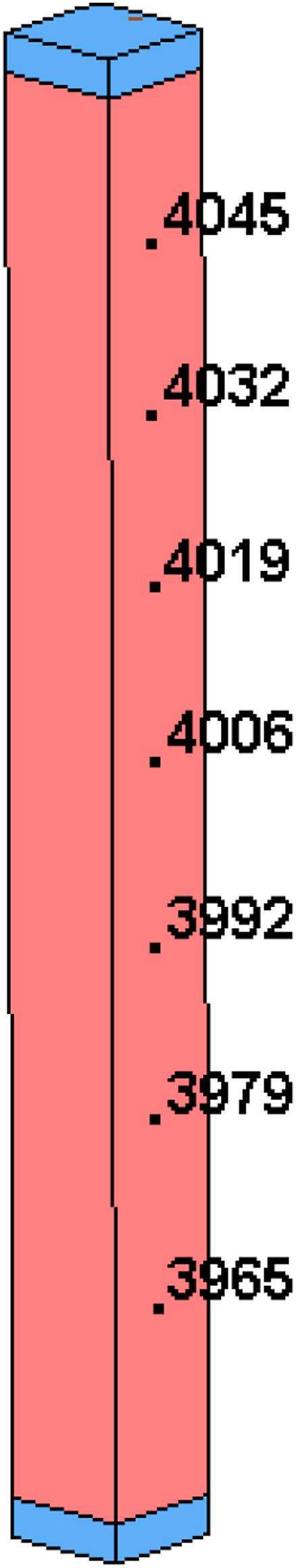
Displacement monitoring points (gauges).

Nodal displacement values were measured and analyzed using ASCII_NODOUT file command with seven monitoring points (gauges) located at 400 mm interval along the length of the column. Figure 8 shows positions of nodal displacement measuring points (gauges) along the length of column.

### 2.7 Erosion criterion

Material damage initiated if further plastic damage occurs after the initial failure surface was reached. LS-DYNA offers a separate element erosion technique and in this study MAT_ADD_EROSION parameters where deployed which gives an option to specify a failure criterion for element erosion up to failure. In this study, the failure criterion was set to have a scalar value (ERODE = 1.05). The model evaluates damage parameter (D) using Eq. (2). The logic behind the damage formulae is the accumulation of increments of effective plastic strain.

(2) $$0\leq \text{D}=\sum \frac{\Delta \varepsilon_{pl}}{\varepsilon_{f}\left(p\right)}\leq 1$$

As it is evident in Eq. (2), erosion of elements is based on the value of the damage parameters, D. If D > 1 and the maximum failure principal strain in the element exceeded the value provided by the user, the element then loses all its stiffness and is deleted from the numerical model leaving the system which simulates the real physical behaviour of the model.

### 2.8 Database

The post-processing unit in finite element analysis (FEA) is a vital stage for yielding and interpreting the results obtained from the already defined pre-processing input files. Nodal displacement values for a given blast scenario was interpreted using ASCII_NODOUT keyword file with 1E−06 small time interval output plotting option is used. In addition to the time interval values each time plot is ordered to be plotted to the current time to determine the next plot.

### 2.9 Mesh sensitivity analysis

**Figure 9:**
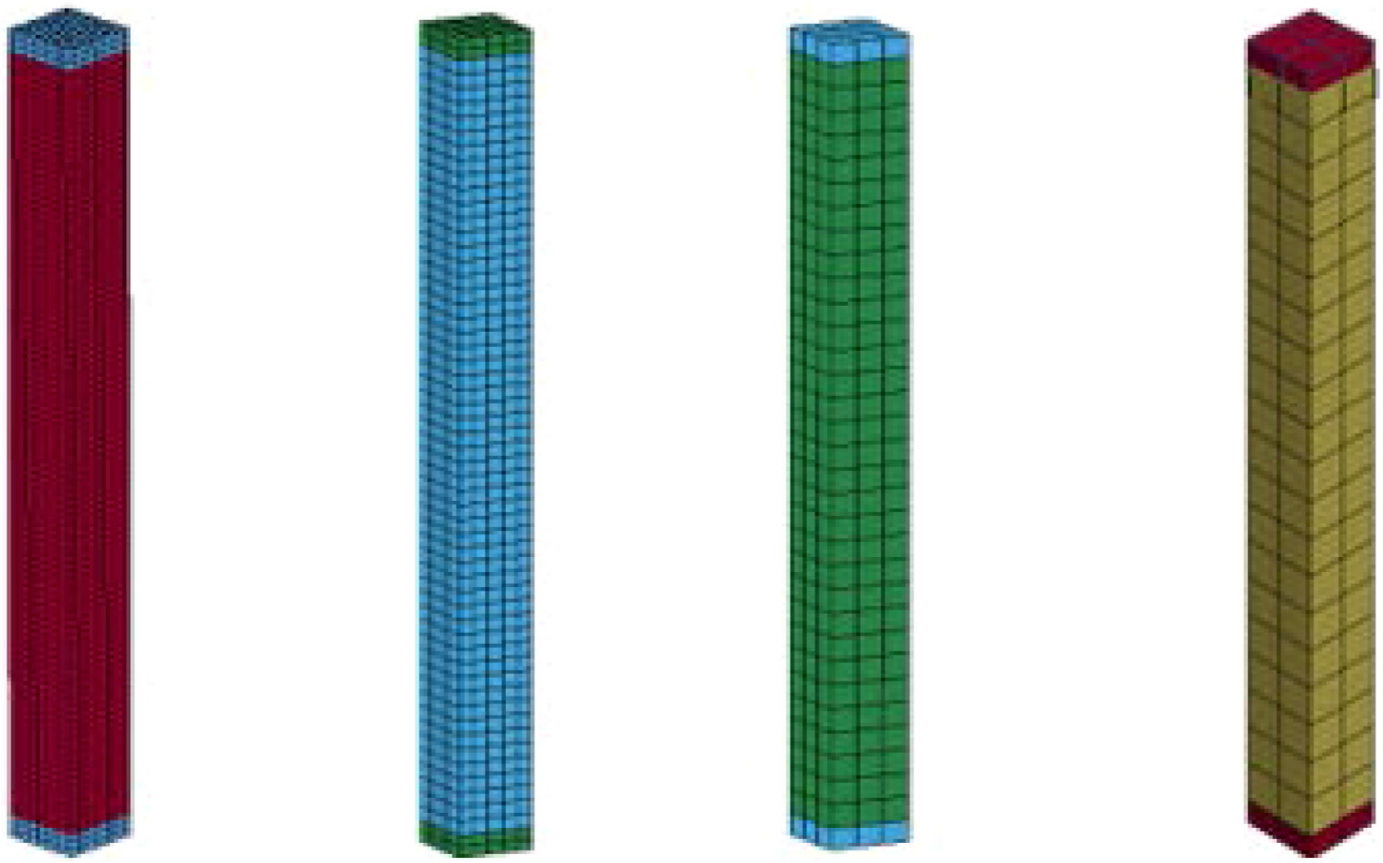
Various LS-DYNA 8-noded hexahedron solid element mesh sizes.

It is obvious that, FEA is an efficient numerical method for solving different type of engineering problems with complicated geometries, loadings and material properties. This all happen if the mathematical and numerical model definitions are good enough. One means of checking the reliability of the numerical model is conducting validation of numerical FE models by field controlled experimental data. In this study, a mesh sensitivity is conducted using a field controlled experimental data provided by Siba [4]. During numerical simulation a 30 mm, 50 mm, 100 mm and 150 mm mesh sizes with same aspect ratios were analyzed. Figure 9 exhibits various LS-DYNA 8-noded hexahedron solid element mesh sizes.

### 2.10 Validation results of FE model

**Figure 10:**
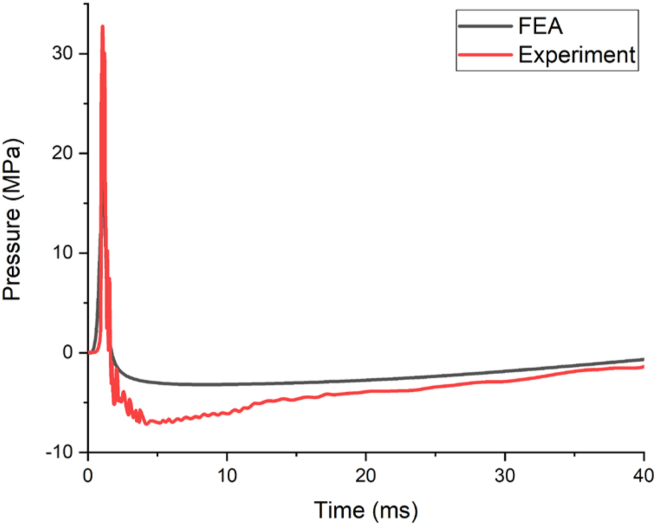
Comparison of pressure-time history.

**Figure 11:**
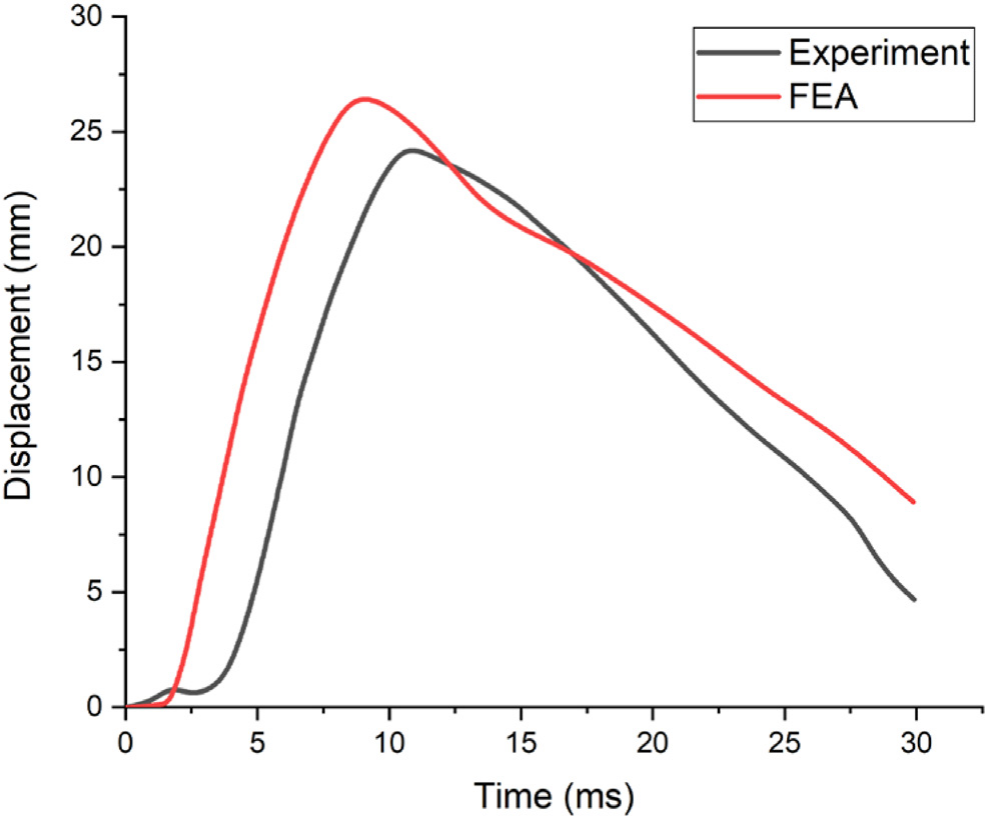
Comparison of displacement-time history.

As part of the experimental program by Siba [4], a conventional column CONV_07 with 300 mm × 300 mm × 3200 mm tested was taken as benchmark experimental data for FE model validation. The RC column was reinforced with 4–25M to give longitudinal reinforcement ratio of 0.02, a concrete cover to reinforcement of 40 mm. The 28-day concrete compressive strength of concrete was 40 MPa while the yield strength of the steel was approximately 400 MPa. Moreover, the blast load parameter was accompanied by a 100 kg ANFO (123 kg TNT) located at 0.65 m height of burst, with 2.50 m stand-off distance. Figures 10 and 11 exhibits a comparison of pressure-time and displacement-time history of experiment and FEA results respectively.

Figure 10 presents the comparison of free-field incident pressure profile of an experimental and FEA results with a peak pressure of 30 MPa was obtained (Figure 10). Moreover, the post-blast field test result revealed that, the maximum displacement of 24.00 mm occurred at lower one-third along the height of the column measured from the bottom support. Whereas, the FEA depicted a maximum displacement of 26.8 mm located on lower one-third along the height of column measured from bottom support (see Figure 11).

**Figure 12:**
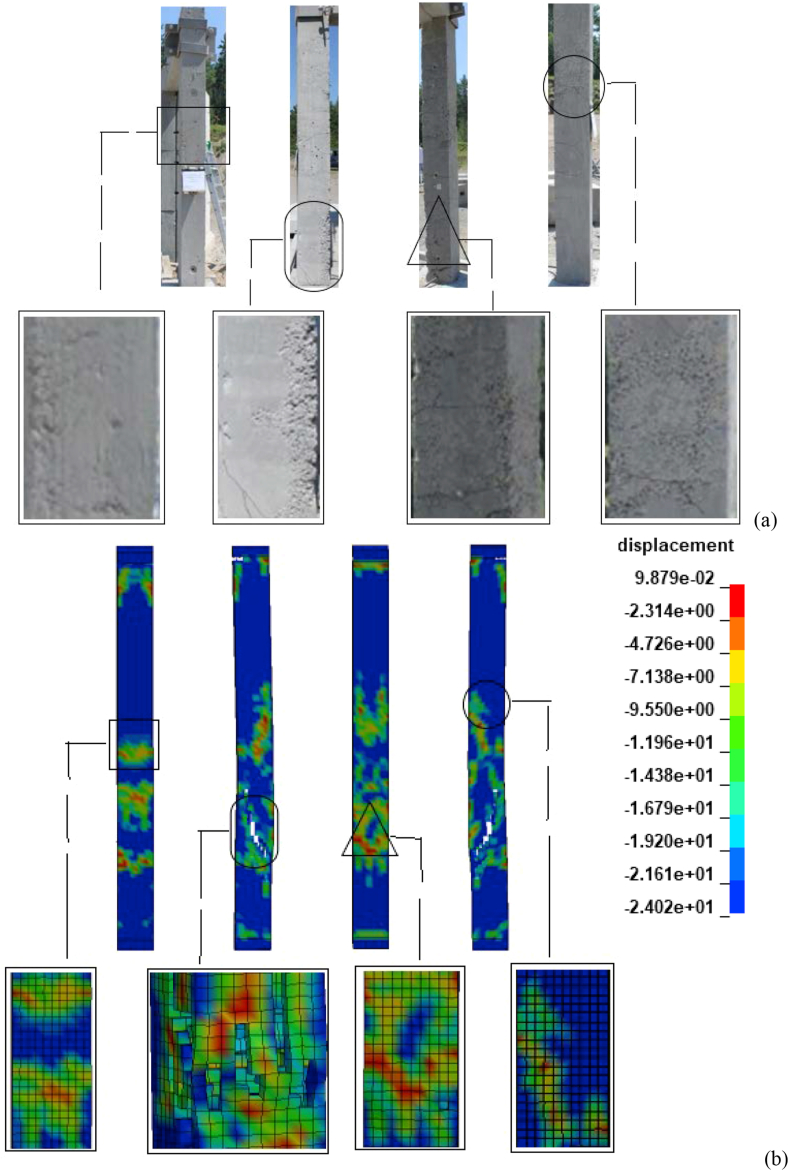
Post-blast column damages: (a) experiment Siba [4]; and (b) FEA.

Figure 12(a) and (b) depicts post-blast column damages for one of the specimens tested in the experiment and FE model. Figure 12(a) exhibits post-test column damage on the column. Specimen's damage evaluation on experimentally tested column after the field blast test illustrated cracking and spalling of concrete cover revealing bending of longitudinal reinforcement in the lower one-third region of the column Siba [4]. As shown in Figure 12(a) minimal flexural cracks were observed on the back-face of the column. Within the lower one-third region, substantial shear cracks were also observed. As shown in Figure 12(b) the numerical simulation after the application of the blast induced shock wave showed a first strike, maximum displacement and respective damage at the lower one-third region of the column as well.

During validation, the total run time of element sizes smaller than 30 mm, made the analysis impractical and it was observed that further decrease in the element size had little effect on numerical results but leads the risk of memory overflow, substantial increase in the computing time, an additional memory to complete solutions, an additional dynamically allocated memory and additional number of CPU. Besides this, numerical analysis with 30 mm mesh size revealed a maximum displacement of 24.00 mm delineated at lower one-third along the height of the column. Likewise, physical observation on experimentally reported column (CONV_07) and FEA model with 30 mm element size illustrated minor concrete spall and small visible residual cracking were observed on the front face, and flexural cracking on the back face. Thus, from the observed displacement and damage values, a 30 mm element size with same aspect ratio revealed best correlation giving adequate accuracy at reasonable run time and is chosen for all subsequent analysis.

## 3 Numerical analysis on effects of constant and variable axial loads

**Figure 13:**
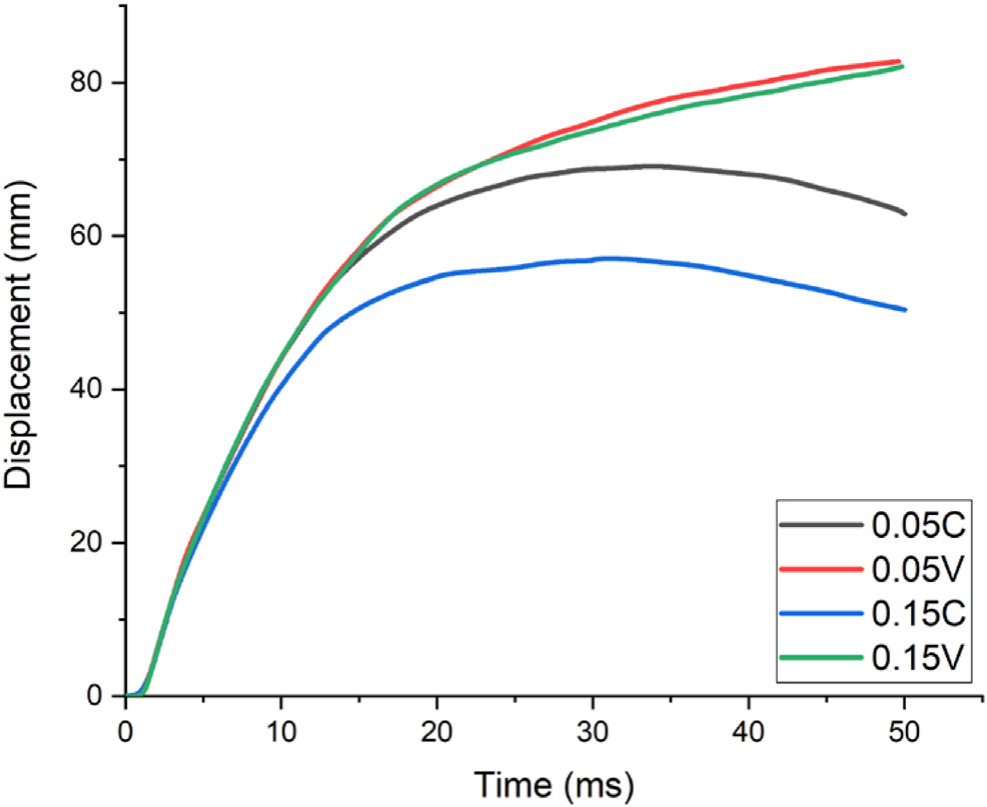
Displacement-time history curves for different constant and variable axial loads with a 0.70 kg/m^3^ scaled distance blast scenario.

**Figure 14:**
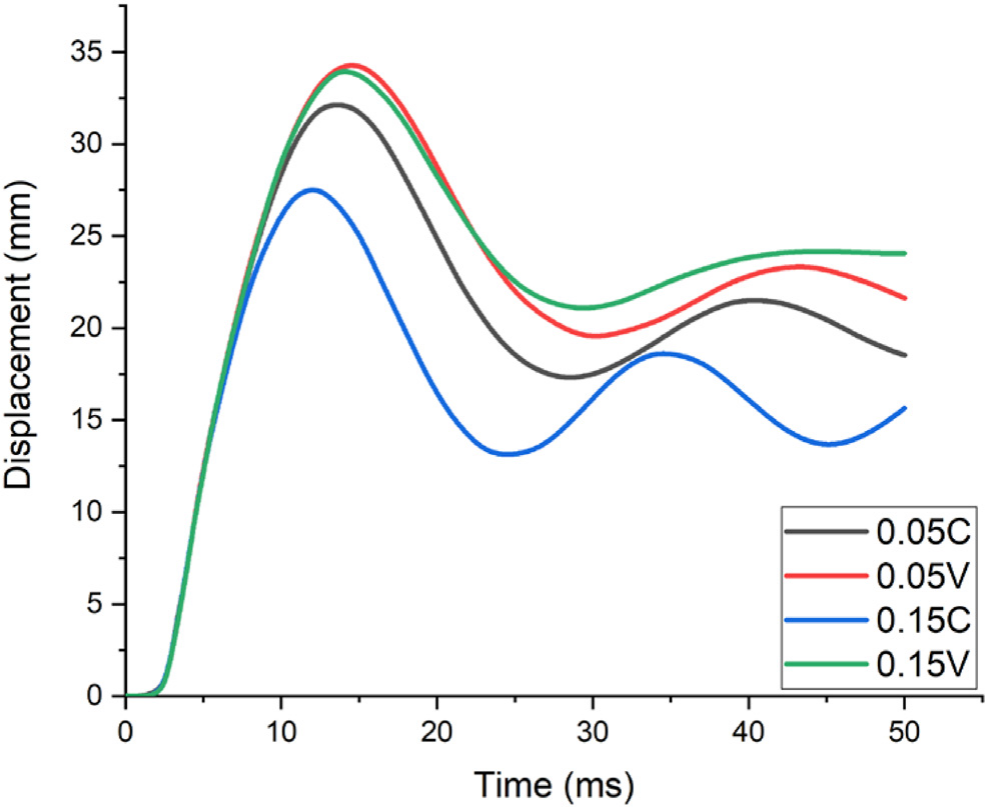
Displacement-time history curves for different constant and variable axial loads with a 1.00 kg/m^3^ scaled distance blast scenario.

**Figure 15:**
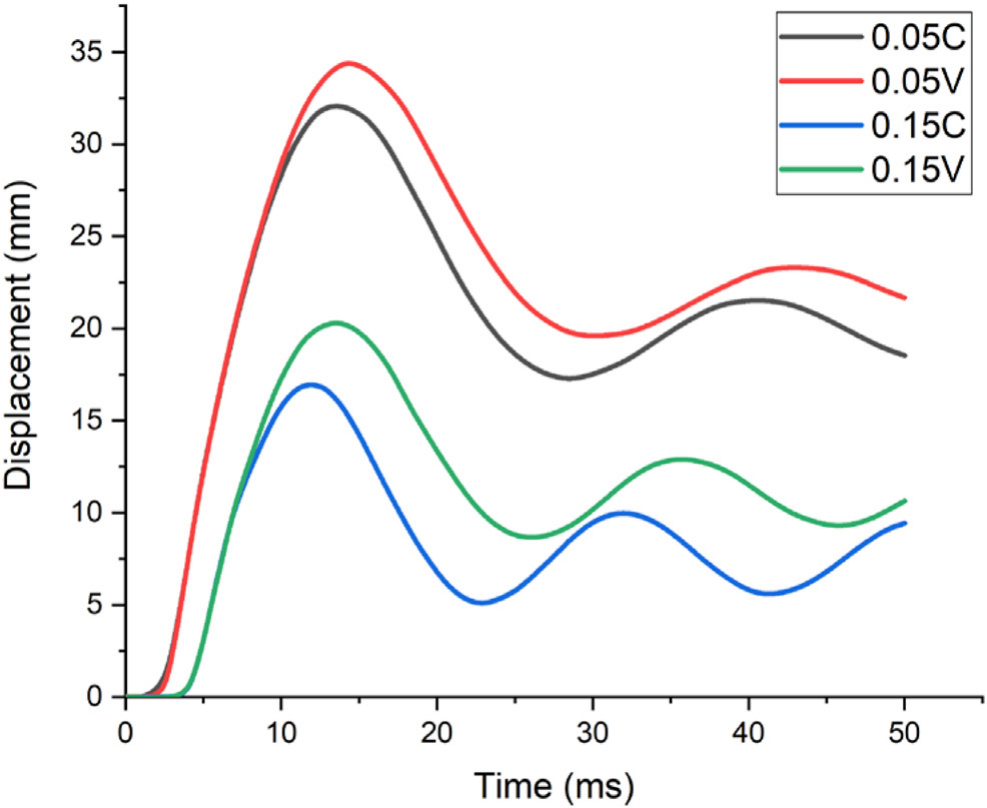
Displacement-time history curves for different constant and variable axial loads with a 1.30 kg/m^3^ scaled distance blast scenario.

The effect of constant and variable axial loads on RC column were studied with 80 kg, 100 kg, 120 kg charge masses yielding scaled distances (Z) of 0.70 kg/m^3^, 1.00 kg/m^3^ and 1.30 kg/m^3^ respectively. The imposed axial loads were computed and derived from the compressive axial load capacity of the RC column. To examine the effect of different levels of compressive axial loads, four axial load ratios (0.05, 0.15, 0.30, and 0.50) were proposed and induced into the system in terms of constant (0.05 C, 0.15 C, 0.30 C, and 0.50 C) and variable axial loads (0.05 V, 0.15 V, 0.30 V, and 0.50 V). Where C and V designated the constant and variable axial loads. The displacement-time history curves of the column are depicted in Figures 13, 14, and 15.

As depicted in Figure 13, for a blast scenario with 80 kg charge mass having a 0.70 m/kg^3^ scaled distance, maximum displacement was found at 1.2 m along height of column. RC column imposed by 0.05 constant and variable axial load ratios (0.05 C and 0.05 V) revealed a maximum nodal displacement of 69.1 mm and 82.8 mm respectively. On the other hand, for the same scaled distance blast scenario, RC column imposed by 0.15 constant and variable axial load ratios (0.15 C and 0.15 V) exhibited a maximum nodal displacement of 57.1 mm and 82.1 mm respectively (see Figure 13).

Consequently, Figure 14 illustrates a result for a blast scenario with 100 kg charge mass having a 1.00 m/kg^3^ scaled distance, maximum displacement was found at 2.0 m along height of column. RC column imposed by 0.05 constant and variable axial load ratios (0.05 C and 0.05 V) had a maximum nodal displacement of 34.4 mm and 36.3 mm respectively. On the other hand, for the same scaled distance blast load case, RC column imposed by 0.15 constant and variable axial load ratios (0.15 C and 0.15 V) exhibited a maximum nodal displacement of 29.5 mm and 35.3 mm respectively (see Figure 14).

Furthermore, Figure 15 depicts a numerical analysis result for a blast scenario with 120 kg charge mass having a 1.30 m/kg^3^ scaled distance, maximum displacement was found at 2.0 m along height of column. RC column imposed by 0.05 constant and variable axial load ratios (0.05 C and 0.05 V) had a maximum nodal displacement of 34.4 mm and 36.4 mm respectively. On the other hand, for the same scaled distance blast load case, RC column loaded by 0.15 constant and variable axial load ratios (0.15 C and 0.15 V) exhibited a maximum nodal displacement of 18.4 mm and 22.1 mm respectively (see Figure 15).

**Figure 16:**
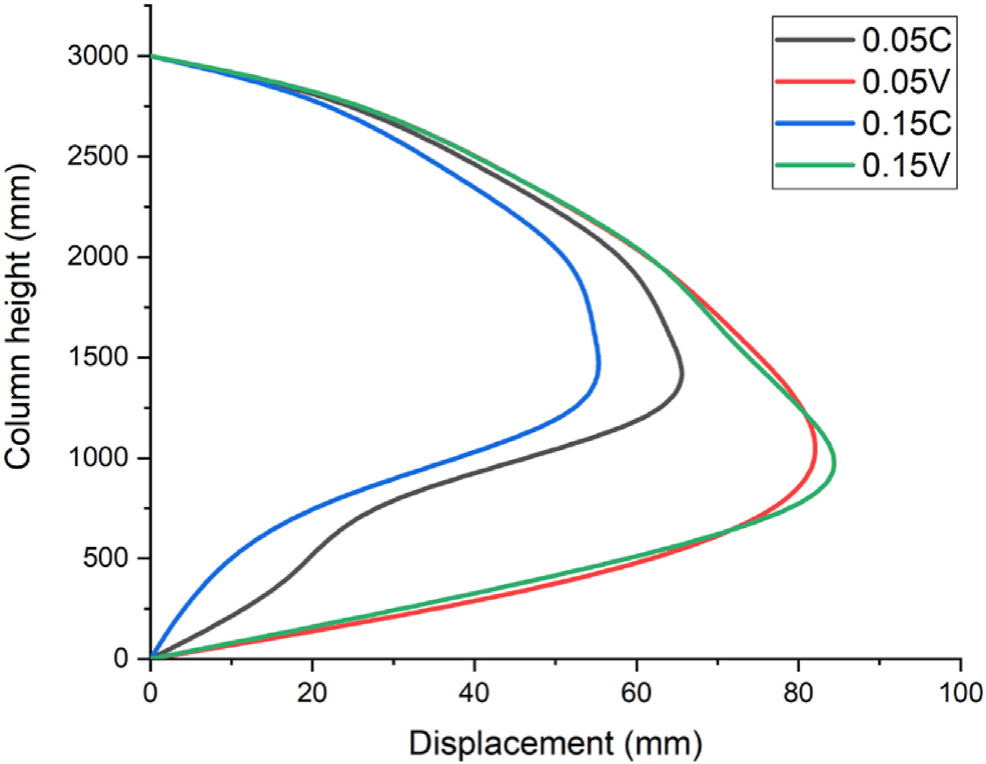
Displacement-time history curves for different constant and variable axial loads with a 0.70 kg/m^3^ scaled distance blast scenario.

**Figure 17:**
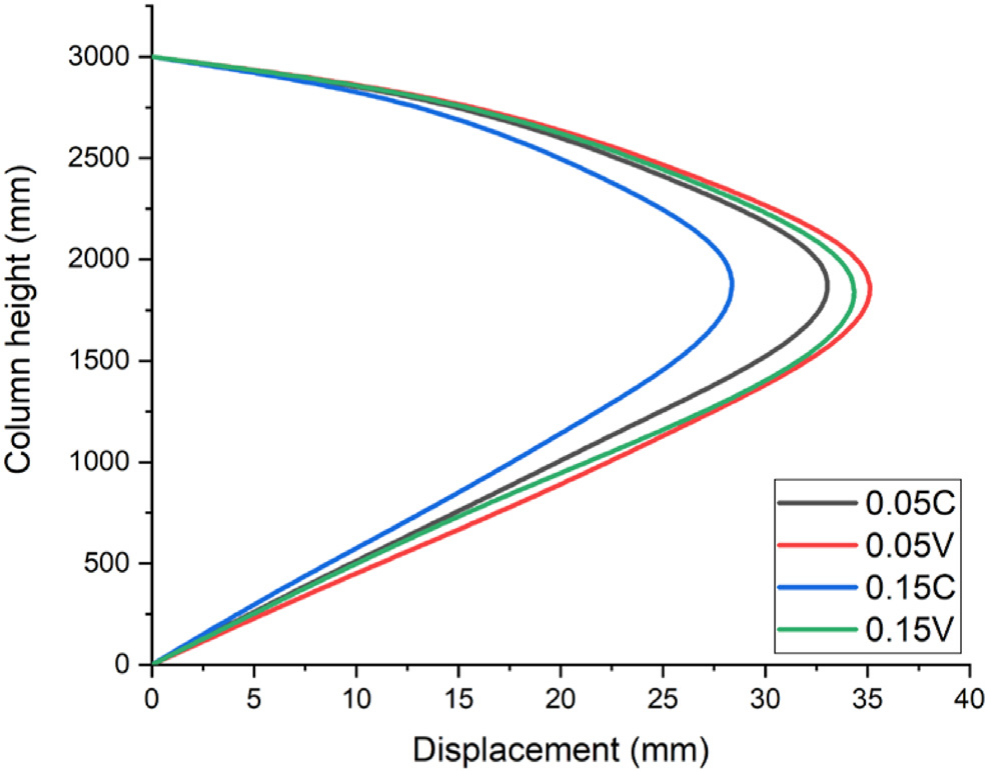
Displacement-time history curves for different constant and variable axial loads with a 1.00 kg/m^3^ scaled distance blast scenario.

**Figure 18:**
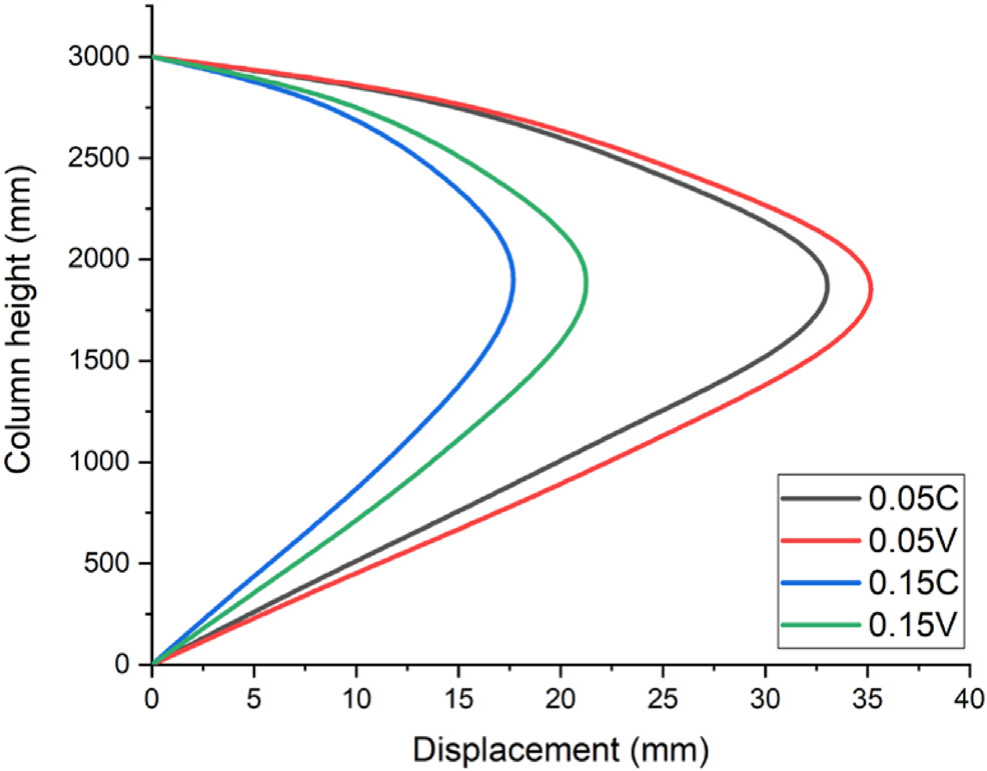
Displacement-time history curves for different constant and variable axial loads with a 1.30 kg/m^3^ scaled distance blast scenario.

In addition to the displacement-time history curves of the column, the effect of constant and variable axial loads on RC column were also studied with three scaled distances (Z) of 0.70 kg/m^3^, 1.00 kg/m^3^ and 1.30 kg/m^3^. The displacement values along the height of column are depicted in Figures 16, 17, and 18. As depicted in Figure 16, for a blast scenario with 0.70 m/kg^3^ scaled distance, RC column imposed by 0.05 constant and variable axial load ratios (0.05 C and 0.05 V) revealed a maximum nodal displacement of 69.1 and 82.7 mm respectively (see Figure 16).

For a close-in blast scenario with scaled distance of 0.70 m/kg^3^, when comparing effect of presence of variable axial load with constant axial load, an increase in the axial load ratio from 0.05 to 0.15 elucidated 17.9 and 7.2 % difference in nodal displacement values.

Likewise, as depicted in Figure 17, for a blast scenario with 1.00 m/kg^3^ scaled distance accompanied by 0.05 constant and variable axial load ratios (0.05 C and 0.05 V) resulted a maximum nodal displacement value of 34.3 and 36.3 mm respectively. But when the axial load ratio was allowed to be increased from 0.05 to 0.15, a decrease in displacement values of 29.5 and 35.2 mm was obtained for reinforced concrete column with constant and variable axial load ratios respectively (see Figure 17).

Figure 18 also exhibits the displacement value of RC column along distinguished height accompanied by a blast scenario of 1.3 m/kg^3^ scaled distance and 0.05 axial load ratio (constant and variable). From the plot, it is evident that, a maximum nodal displacement of 34.3 and 36.3 mm was computed. In contrary, while increasing the axial load ratio from 0.05 to 0.15, a significant drop in displacement of 18.3 and 22.1 mm was obtained revealing 46.6 and 39.4 % difference in nodal displacement respectively (see also Figure 18).

**Figure 19:**
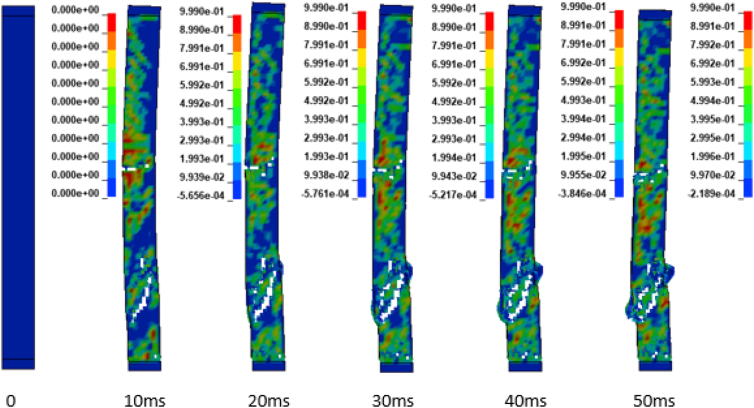
Damage profile for RC column with 0.05 constant axial load ratio accompanied by 0.70 kg/m^3^ scaled distance blast scenario.

**Figure 20:**
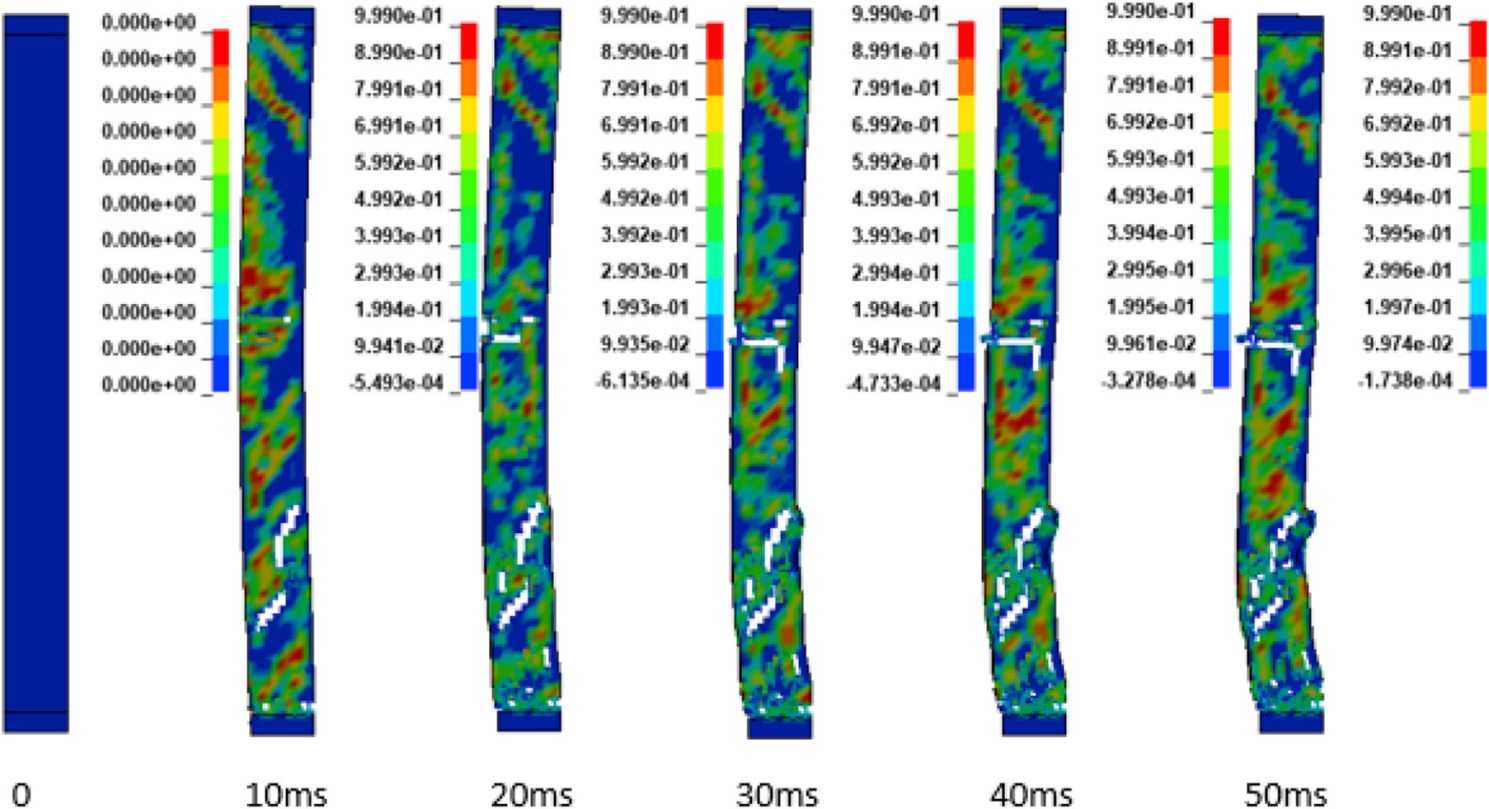
Damage profile for RC column with 0.05 variable axial load ratio accompanied by 0.70 kg/m^3^ scaled distance blast scenario.

**Figure 21:**
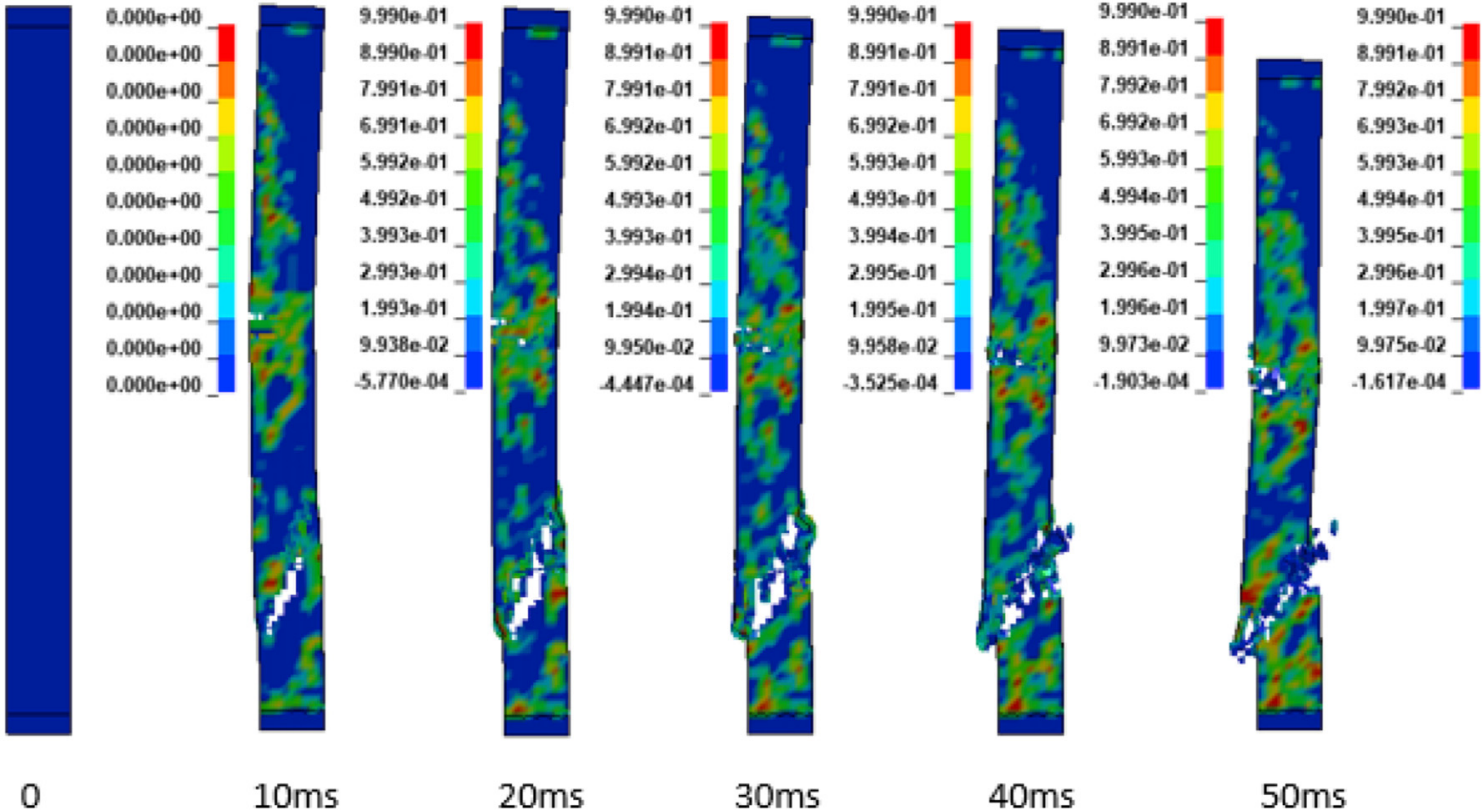
Damage profile for RC column with 0.15 constant axial load ratio accompanied by 0.70 kg/m^3^ scaled distance blast scenario.

**Figure 22:**
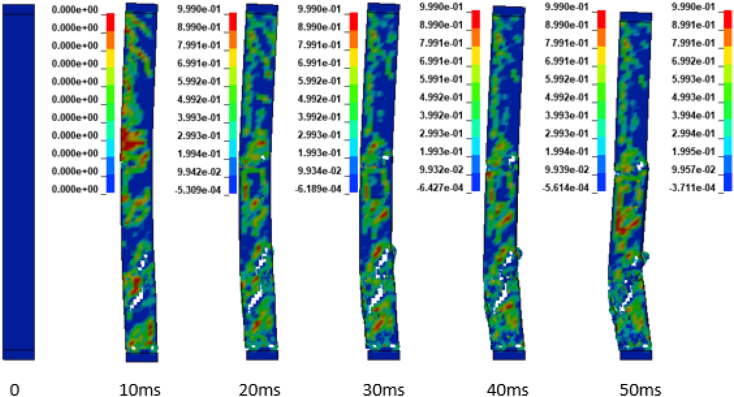
Damage profile for RC column with 0.15 variable axial load ratio accompanied by 0.70 kg/m^3^ scaled distance blast scenario.

**Figure 23:**
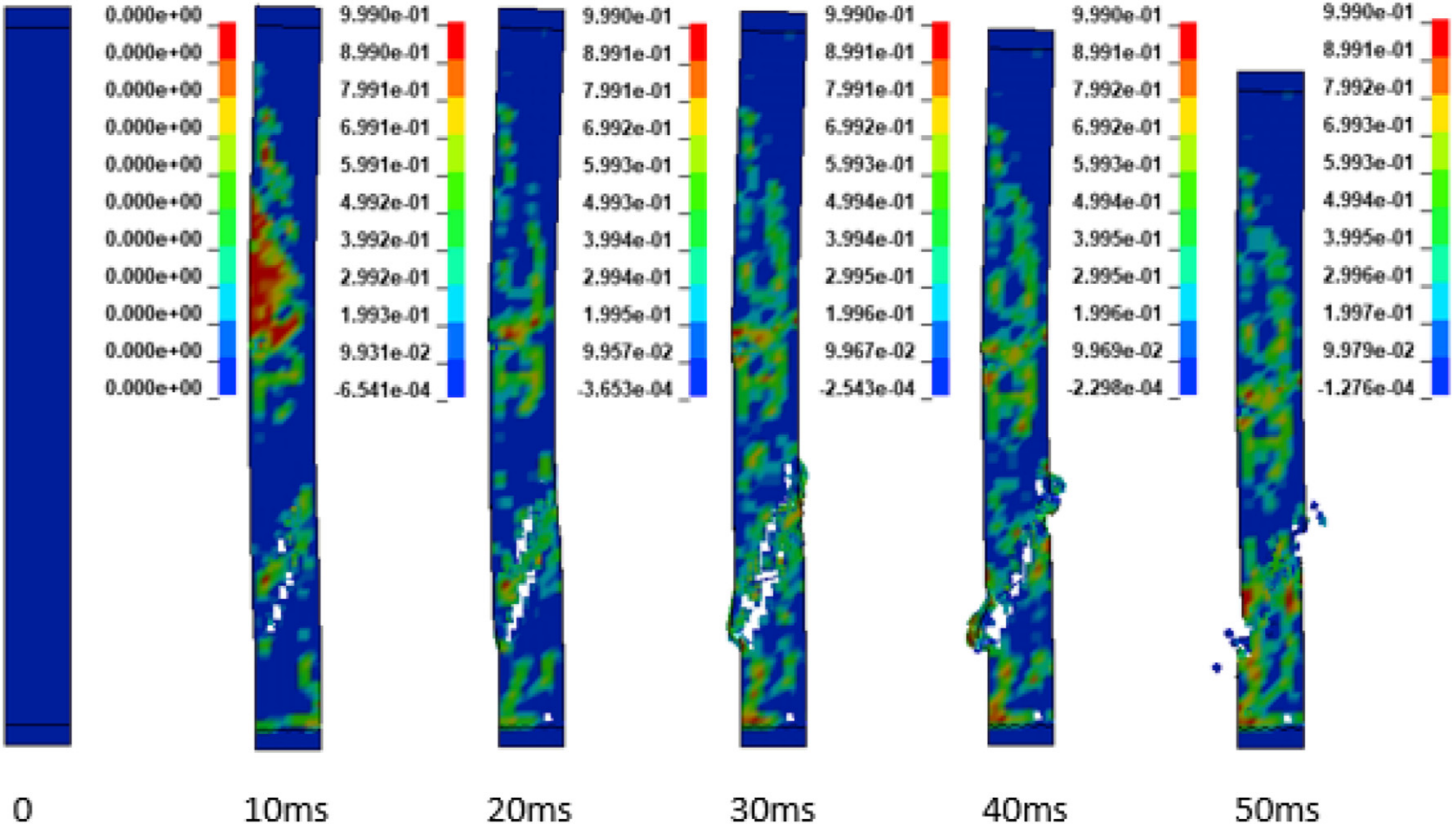
Damage profile for RC column with 0.30 constant axial load ratio accompanied by 0.70 kg/m^3^ scaled distance blast scenario.

**Figure 24:**
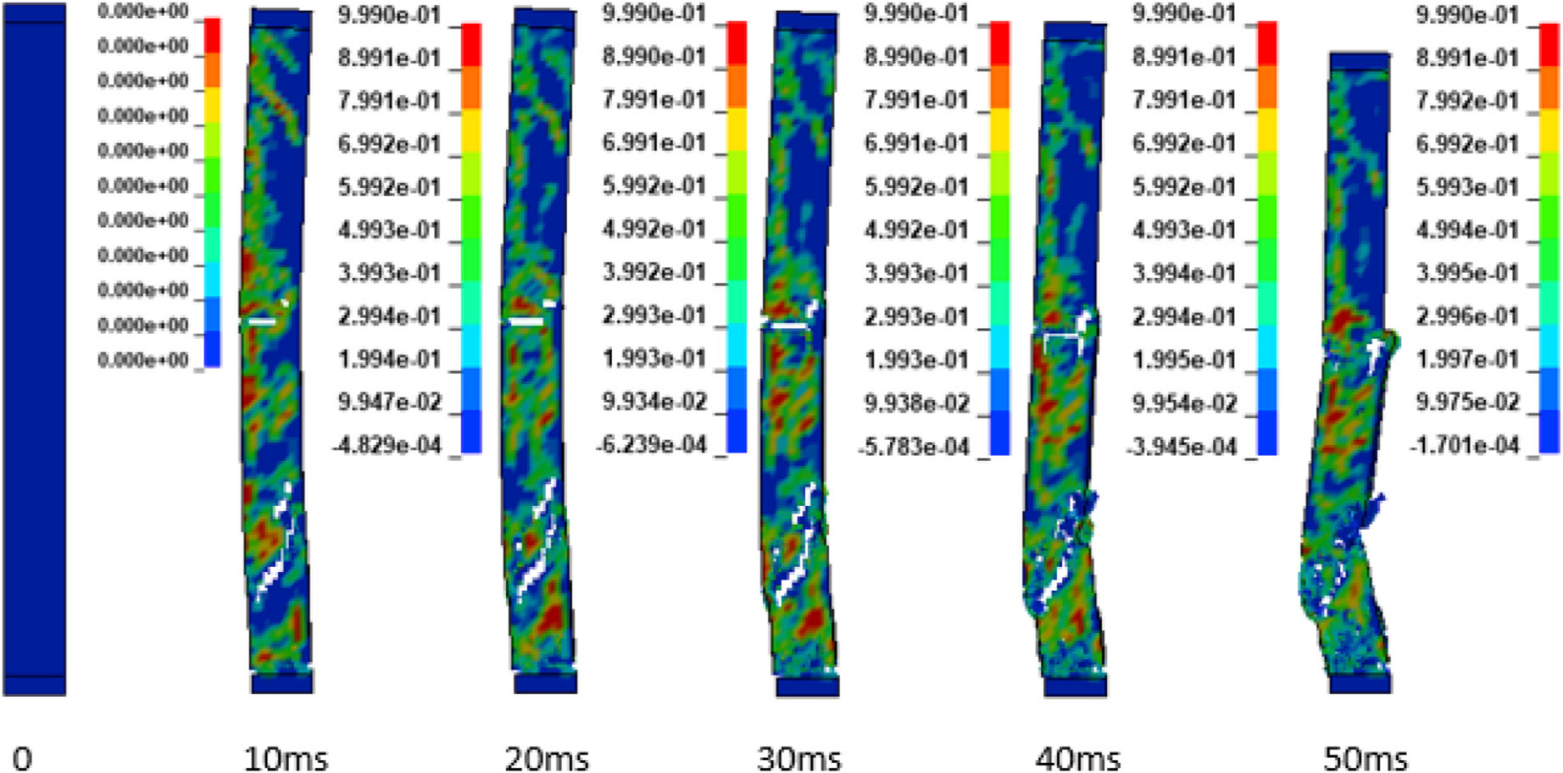
Damage profile for RC column with 0.30 variable axial load ratio accompanied by 0.70 kg/m^3^ scaled distance blast scenario.

**Figure 25:**
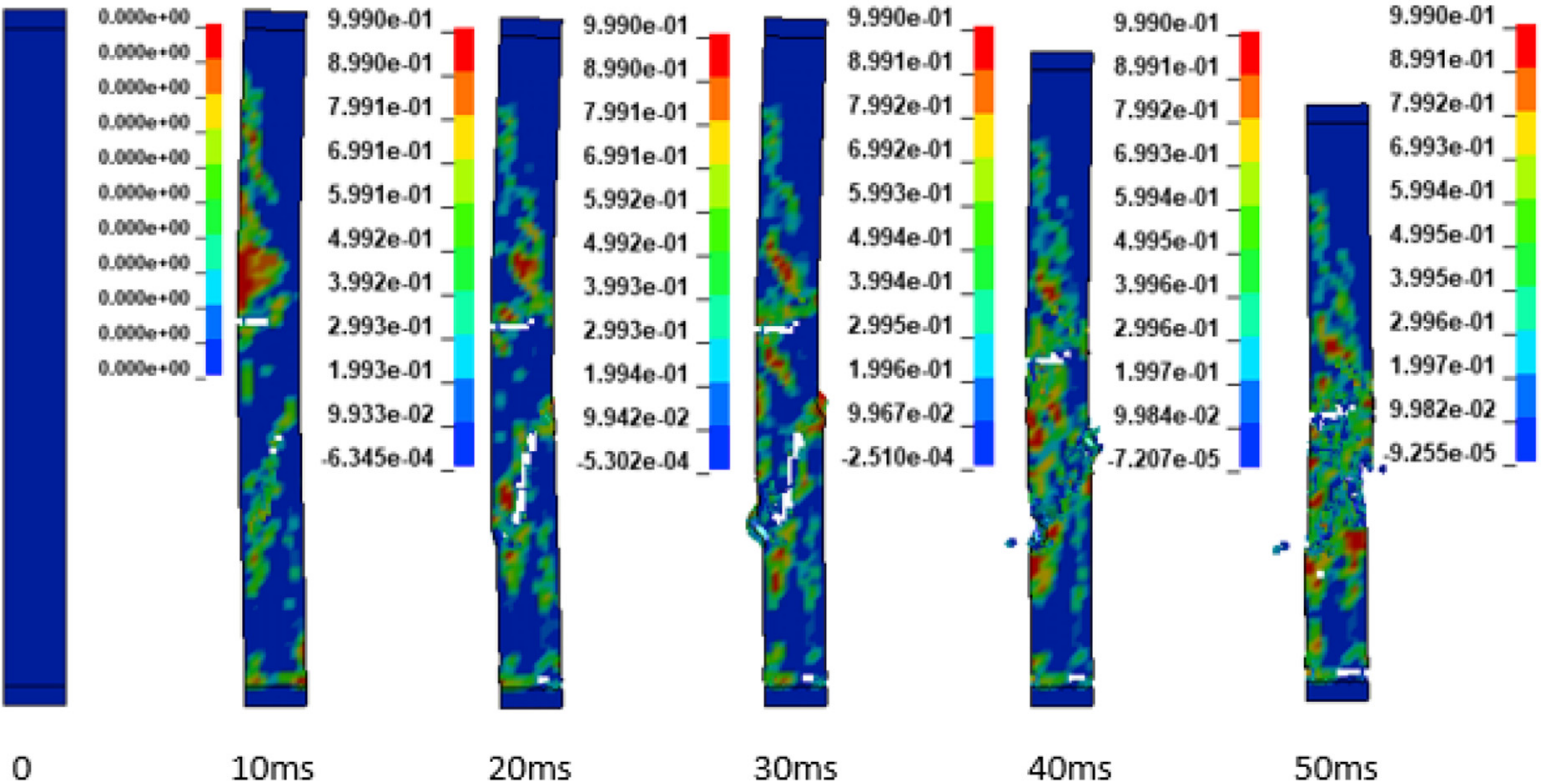
Damage profile for RC column with 0.50 constant axial load ratio accompanied by 0.70 kg/m^3^ scaled distance blast scenario.

**Figure 26:**
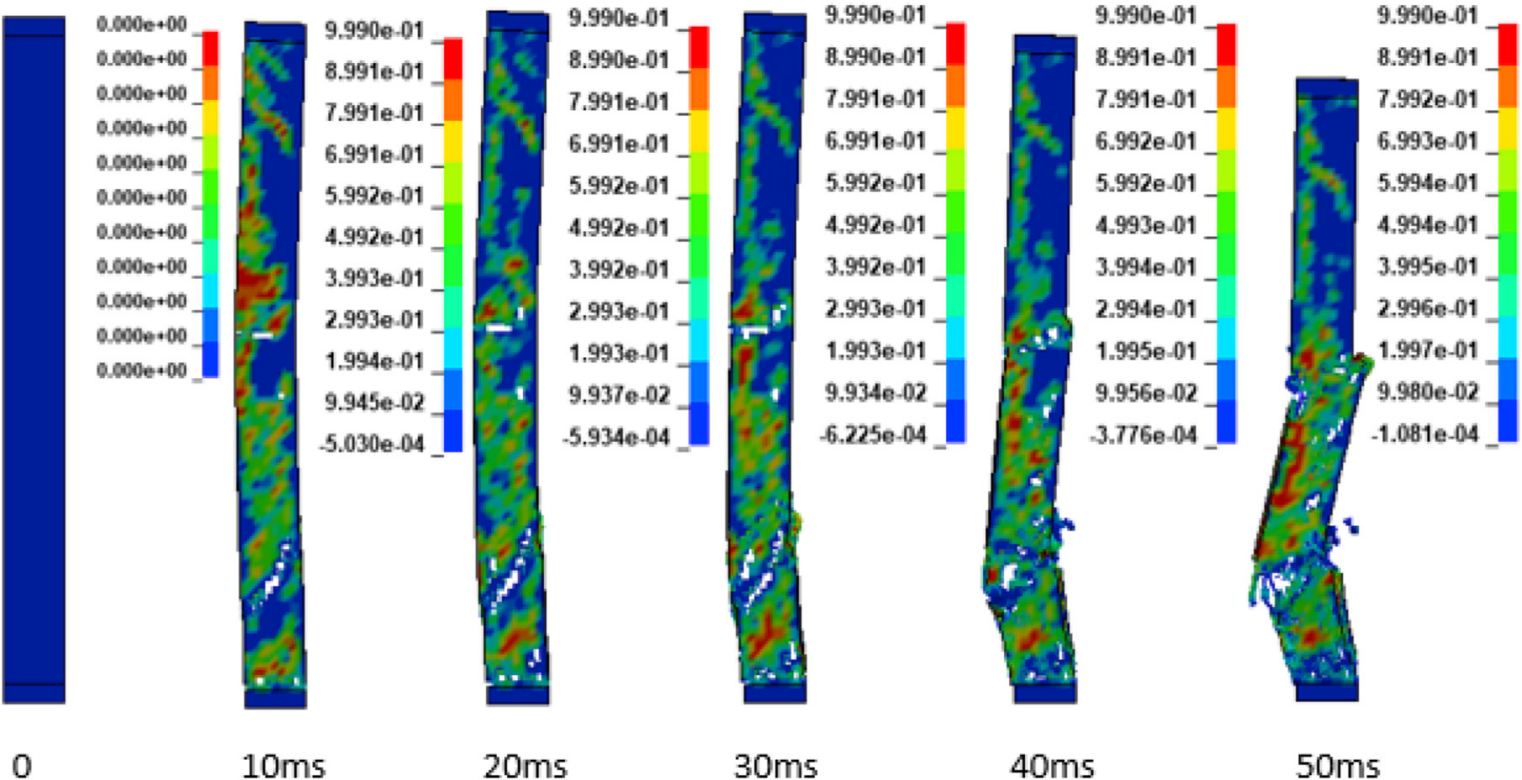
Damage profile for RC column with 0.50 variable axial load ratio accompanied by 0.70 kg/m^3^ scaled distance blast scenario.

In addition to the displacement values of the study RC column, the extent damage of the column is also evaluated by the use of effective plastic strain damage profile (Figures 19, 20, 21, 22, 23, 24, 25, and 26). A total of four different axial load ratios (0.05 C, 0.05 V 0.15 C, 0.15 V, 0.3 C, 0.3 V, and 0.5 C, 0.5 V) were used to study the damage propagation and extent of a 0.7 kg/m^3^ scaled distance blast induced shock wave loading cases.

Figures 19 and 20 depicts a direct shear failure damage profile for the reinforced concrete column at scaled distance of 0.7 m/kg^3^ with 0.05 C and 0.05 V respectively during hemispherical surface type of burst. Since the blast explosive charge mass was located on the ground, a shear failure mode on reinforced concrete column is observed to be first initiated at bottom support and then propagates to the rest part of the column. The local shear failures especially crushing of concrete and bending of reinforcing steel bars were observed to be dominant at the exact location of bottom and mid points of the column.

Figures 21 and 22 exhibits a direct shear failure damage profile for the reinforced concrete column at scaled distance of 1.00 m/kg^3^ with 0.15 C and 0.15 V respectively. For RC column loaded with a 0.15 C revealed a spontaneous shear failure typically designated by a crushing of concrete and breakage of reinforcing steel bars. On the other hand, for a blast scenario on the RC column with 0.15 V, the local shear failures especially crushing of concrete and bending of reinforcing steel bars were observed to be visible in progressive manner.

Figures 23, 24, 25, and 26 demonstrates damage profiles on the RC column loaded with scaled distance of 0.70 m/kg^3^ with both 0.30 C, 0.30 V, 0.50 C and 0.50 V resulted a large pressure with nonlinear pressure distribution caused the reinforced concrete column in fracture states of failure encompassing disintegration of concrete core yielding complete crushing of concrete from the column and sever buckling resulting breakage of longitudinal and transverse steel bars was observed.

## 4 Conclusion

In this study, the effect of constant and variable axial loads on a reinforced concrete column under different scaled distance blast scenarios were investigated. A nonlinear FEA software (LS-DYNA) was employed and total of four axial load ratios of 0.05, 0.15, 0.30, and 0.50 were imposed into the column. After conducting a nonlinear dynamic analysis on the reinforced concrete columns the displacement-time history, displacement along the height of column, and effective plastic strain damage profiles were extracted and the following major concluding remarks are made:

• An increase in axial load ratios from 0.05 to 0.5 accompanied by a small scaled distance blast scenario (0.70 kg/m^3^) made the reinforced concrete column to suffer severe damages including crushing of concrete especially direct shear failures and breaking of reinforcing steel bars.
• Comparing the nature and behaviour of variable axial loads with constant axial loads, the former loading case revealed larger nodal displacement values along height of column, and a higher displacement-time history curve was traced. In addition to this, the damage propagation nature of RC columns loaded with variable axial load was progressive while RC columns with constant axial loads accompanied by small scaled blast scenario, the initiation and propagation was rendered to have a severely crushed concrete element without bending actions leaving the entire column inadequate for service.

Finally, this study is limited to reinforced concrete columns designed for only constant and variable gravity loads, it is worth to forward a future study needs on investigation of effects of blast load on a reinforced concrete column strengthened by different reinforcement and retrofitting techniques.

## Declarations

### Author contribution statement

Solomon Abebe: Conceived and designed the experiments; Performed the experiments; Analyzed and interpreted the data; Wrote the paper.

Solomon Getachew: Analyzed and interpreted the data; Wrote the paper.

### Funding statement

This research did not receive any specific grant from funding agencies in the public, commercial, or not-for-profit sectors.

### Data availability statement

Data will be made available on request.

### Declaration of interest's statement

The authors declare no conflict of interest.

### Additional information

No additional information is available for this paper.
